# Perioperative Angiotensin-(1-7) for Postoperative Cognitive Vulnerability Following Coronary Artery Bypass Surgery: A Pilot Case Series

**DOI:** 10.21203/rs.3.rs-9296663/v1

**Published:** 2026-05-12

**Authors:** Christina Hoyer-Kimura, John Konhilas, Lee Ryan, Nancy K Sweitzer, David Bull, Meredith Hay

**Affiliations:** University of Arizona; University of Arizona; University of Arizona; Washington University School of Medicine; University of Arizona; University of Arizona

**Keywords:** postoperative cognitive impairment, perioperative cognition, coronary artery bypass graft, angiotensin-(1-7), neurovascular unit, neurofilament light chain, cognitive vulnerability, cardiac surgery

## Abstract

**Background:**

Postoperative cognitive impairment is a common complication following coronary artery bypass grafting (CABG) and is thought to arise from perioperative neurovascular injury, systemic inflammation, and neuroaxonal damage. Despite its clinical impact, there are no approved pharmacologic strategies to mitigate postoperative cognitive vulnerability. Angiotensin-(1-7) (Ang-(1-7); PNA1), a Mas receptor agonist, has demonstrated neurovascular and anti-inflammatory effects in preclinical models, but its perioperative cognitive effects in humans have not been described.

**Case presentation:**

This case series reports outcomes from an incompletely accrued randomized, double-blind, placebo-controlled pilot study in older adults undergoing elective CABG. Five participants (mean age 64.6 years; 80% male) completed all study procedures; three received Ang-(1-7) (200 μg/kg/day subcutaneously) and two received saline as the placebo. Study drug administration was initiated intraoperatively within two hours of chest opening and continued daily for 21 days. Cognitive performance was assessed at baseline and 21 days postoperatively using standardized neuropsychological measures, including face–name associative memory and executive function tasks. Neuroaxonal injury was evaluated using plasma neurofilament light chain (NfL) measured by ultrasensitive single-molecule array assays. Perioperative glucose regulation, blood pressure, renal function, and adverse events were monitored for safety. Participants receiving Ang-(1-7) demonstrated relative preservation of associative memory and attenuation of postoperative NfL levels compared with placebo-treated participants, with no drug-relted adverse events or clinically significant renal or hemodynamic complications observed.

**Conclusions:**

In this small perioperative case series, Ang-(1-7) administration following CABG was feasible and well tolerated and was associated with signals of cognitive preservation and reduced neuroaxonal injury during early postoperative recovery. These findings support further investigation of Ang-(1-7) as a potential perioperative strategy to mitigate postoperative cognitive vulnerability in patients undergoing cardiac surgery.

## Background

Postoperative cognitive impairment remains a common and clinically significant complication following cardiac surgery, particularly coronary artery bypass grafting (CABG). Cognitive impairment has been reported in approximately 30–60% of patients in the early postoperative period following coronary artery bypass grafting, with persistent deficits observed in 10–30% of patients at three to six months after surgery (1), (2), (3), (4).

Although the long-term contribution of surgery itself versus underlying cerebrovascular and neurodegenerative vulnerability remains debated, perioperative insults—including systemic inflammation, cerebral hypoperfusion, blood–brain barrier (BBB) disruption, and neurovascular unit (NVU) dysfunction—are thought to play an important role in early postoperative cognitive trajectories (5), (6).

Emerging biomarker data supports the concept that perioperative neuronal injury contributes to postoperative cognitive outcomes. Neurofilament light (NfL)- a cytoskeletal protein released into cerebrospinal fluid and blood following axonal injury- has been validated as a sensitive peripheral marker of neuronal damage across neurodegenerative, inflammatory, and perioperative contexts (7), (8). In patients undergoing cardiac surgery, perioperative increases in plasma NfL have been associated with delirium and longer-term cognitive decline, while higher baseline NfL levels reflect preexisting neuronal vulnerability (9), (10), (11). In a large prospective cohort of cardiac surgery patients, Brown et al. demonstrated that increases in plasma NfL on postoperative day 1 were associated with worse cognitive outcomes at one year, supporting the biological relevance of early perioperative neuronal injury to downstream cognitive trajectories (12).

Despite this growing mechanistic understanding, there are currently no approved pharmacologic interventions specifically targeting perioperative neuronal or neurovascular injury to mitigate postoperative cognitive impairment. Angiotensin-(1–7) (Ang-(1–7)), an endogenous peptide of the non-classical renin–angiotensin system, has emerged as a candidate neurovascular protective agent. Acting primarily through the Mas receptor (MasR), Ang-(1–7) exerts vasodilatory, anti-inflammatory, endothelial-stabilizing, and BBB–protective effects that directly counteract mechanisms implicated in perioperative cognitive dysfunction (13), (14). Preclinical studies have demonstrated that Ang-(1–7) improves cerebral perfusion, preserves BBB integrity, reduces neuroinflammation, decreases NfL, and attenuates cognitive deficits in models of vascular cognitive impairment and neurovascular injury (14), (15), (16), (17).

These mechanistic properties suggest that perioperative administration of Ang-(1–7) may provide neurovascular and neuroaxonal protection during the high-risk surgical period, potentially attenuating early postoperative cognitive vulnerability rather than reversing established neurodegeneration. This concept aligns with emerging views that perioperative cognitive trajectories may be modified by interventions targeting acute NVU stress and neuronal injury, particularly in patients with underlying cerebrovascular disease or reduced cognitive reserve (18), (19).

Here, we report a small exploratory case series from an investigator-initiated randomized, double-blind, placebo-controlled study evaluating perioperative Ang-(1–7) administration in older adults undergoing elective CABG. Although originally designed as a larger trial, operational constraints limited completion to five participants. This report focuses on descriptive cognitive trajectories, plasma NfL measurements, and safety outcomes through the early postoperative period. Consistent with the exploratory nature of this case series, results are presented to generate hypotheses regarding perioperative neurovascular and cognitive protection rather than to establish efficacy.

### Case presentation

## Methods

### Study Design and Oversight

All participants were recruited from Banner University Health Science Center (Tucson, Arizona). The study was conducted in accordance with the Declaration of Helsinki and Good Clinical Practice guidelines and was approved by the Western Institutional Review Board. Written informed consent was obtained from all participants prior to study initiation.

This report describes a small, descriptive case series derived from an investigator-initiated, randomized, double-blind, placebo-controlled clinical study evaluating the safety and potential cognitive effects of perioperative Ang-(1–7) administration in older adults undergoing elective CABG. The original protocol was designed as a larger randomized clinical trial; however, enrollment was terminated early due to operational constraints, and only five participants completed the perioperative study procedures relevant to this analysis. The present report is therefore exploratory and hypothesis-generating, with a specific focus on early post-operative cognitive and physiological outcomes.

Baseline cognitive testing and blood-based biomarkers were obtained during the preoperative screening period (1–15 days before CABG), and at follow-up on Day 21 post-surgery. Blood biomarkers from CABG participants were compared with healthy, age- and sex-matched samples from the Precision Medicine True Normal Biobank (Precision Medicine Group, LLC).

### Participants

Eligible participants were adults aged 60–80 years undergoing first-time, elective, on-pump CABG surgery. Inclusion criteria included a baseline Mini-Mental State Examination (MMSE) score ≥ 25 and the ability to complete neuropsychological testing. Exclusion criteria included recent cerebrovascular events, known neurodegenerative disease, major psychiatric illness, significant renal or hepatic dysfunction, active malignancy, or use of investigational agents within 30 days prior to enrollment.

### Randomization and Blinding

Participants were randomized in a 2:1 ratio to receive Ang-(1–7) or placebo using site-specific block randomization procedures implemented by unblinded investigational pharmacy personnel not involved in clinical assessments. Study drug preparation and dispensing were performed by the investigational pharmacy. Participants, investigators, outcome assessors, and clinical staff remained blinded to treatment allocation throughout the perioperative study period.

### Investigational Product and Administration

Ang-(1–7) was supplied as a GMP-manufactured lyophilized peptide and administered via subcutaneous injection at 200 μg/kg/day for 21 consecutive days. The initial dose was administered intraoperatively within two hours of chest opening. Subsequent doses were administered daily during hospitalization by trained clinical staff and, following discharge, by the participant or caregiver after standardized injection training and competency verification. Placebo consisted of sterile saline administered using identical procedures.

### Cognitive and Neuropsychological Assessments

Participants underwent standardized neuropsychological assessment at baseline during the preoperative screening period (1–15 days before CABG) and at Day 21 following surgery, corresponding to the early post-operative recovery period. The cognitive battery was selected to assess domains commonly affected by perioperative cognitive dysfunction and vascular-related cognitive vulnerability, including episodic and associative memory, executive function, attention, processing speed, and cognitive control.

The assessment battery included the Consortium to Establish a Registry for Alzheimer’s Disease (CERAD) Word List and Rey Auditory Verbal Learning Test (RAVLT) to assess verbal learning, delayed recall, and interference; Trail Making Test (TMT, Parts A and B) to assess processing speed and executive set-shifting; the Face–Name Associative Memory Examination (FNAME) including Cued Name Recall (CRN) to assess associative episodic memory; Stroop Color–Word paradigms to assess inhibitory control; and reaction time–based tasks to assess attentional vigilance and processing speed. All assessments were administered according to standardized protocols by trained personnel blinded to treatment assignment.

The cognitive battery generated multiple task-specific performance metrics, including accuracy, reaction time, and derived executive control measures. For attention and executive function tasks, outcomes included condition-specific accuracy and reaction time (e.g., congruent, incongruent, and neutral conditions), as well as global and local shift costs where applicable. For memory assessments, both performance scores (e.g., items recalled, recognition accuracy) and error metrics (e.g., free recall intrusion errors) were recorded. Reaction time tasks yielded measures of accuracy, central tendency (mean and median reaction time), and variability. Given the exploratory nature of this case series, analyses focused on domain-level patterns rather than individual subscale comparisons.

Delirium during hospitalization was assessed using the Confusion Assessment Method (CAM). Mood and suicidality were monitored using validated instruments, including the Geriatric Depression Scale or Beck Depression Inventory and the Columbia Suicide Severity Rating Scale.

### Blood Biomarker Neurofilament Light (NfL)

Serum samples of age-matched controls (provided from Precision Medicine True Normal Biobank) and CABG patients obtained at baseline and follow-up visits were used for NfL assessment. Baseline samples were collected in CABG participants at the preoperative screening visit (1–15 days before CABG), and follow-up samples were collected on Day 21 post-surgery. All samples were stored at −80 C before shipment to PBL Assay Science for measurement of NfL. NfL was measured using a Quanterix-Simoa assay (SimoaTM NF-Light^®^ kit: Quanterix # 103186, PBL, Neurofilament-Light Advantage Assay Kit). Samples were run in duplicate. Analyses focused on patterns of NfL levels rather than individual subscale comparisons.

### Blood Pressure Monitoring

Blood pressure (BP) was monitored to assess hemodynamic safety during perioperative administration of Ang-(1–7). Measurements were obtained before study drug injection and then hourly for six hours following dosing while the participant remained in the inpatient setting. Mean arterial pressure (MAP) values were recorded and reviewed for clinically significant deviations. BP was monitored outside this window according to standard postoperative clinical care practices throughout hospitalization. Comparisons were descriptive and focused on identifying any treatment-associated hypotension or hemodynamic instability.

### Laboratory Assessments

Routine perioperative laboratory assessments were obtained as part of standard clinical care and safety monitoring. The values were obtained at baseline (7–14 days pre-surgery), on the day of surgery, and daily for 4 days post-surgery. These included complete blood counts (hemoglobin, hematocrit, red and white blood cell counts, and platelets), metabolic panels (electrolytes, renal function markers, and glucose), liver function tests, protein indices (including serum albumin and total protein), and coagulation parameters. Laboratory values were reviewed for clinically significant abnormalities and treatment-emergent changes. Given the exploratory nature of this case series, analyses focused on identifying clinically meaningful safety signals rather than formal statistical comparison of individual laboratory measures.

### Renal Function Monitoring

Renal function was monitored perioperatively using serial measurements of serum creatinine, blood urea nitrogen (BUN), and calculated BUN/creatinine ratio as part of routine clinical laboratory assessments. Renal function was summarized using the estimated glomerular filtration rate (eGFR; CKD-EPI 2021 race-free equation) derived from serum creatinine. The values were obtained at baseline (7–14 days pre-surgery), on the day of surgery, and daily for 4 days post-surgery.

### Safety Monitoring

Adverse events (AEs) and serious adverse events (SAEs) were collected from the time of informed consent through the Day 21 follow-up visit. Events of special interest included hypotension, thromboembolic events, cerebrovascular events, hypoglycemia or hyperglycemia, and clinically significant changes in mood or suicidality. Safety assessments were conducted during hospitalization and through scheduled follow-up contact during the perioperative period. All SAEs were reported in accordance with IRB and FDA requirements.

### Statistical Analysis

Given the small sample size, all analyses were descriptive. Cognitive and physiological outcomes are presented as individual participant trajectories and qualitative group-level trends comparing baseline and Day 21 performance without formal hypothesis testing. This approach was selected to characterize feasibility, safety, and potential early signals of perioperative cognitive or neuroaxonal protection to inform future adequately powered clinical studies.

## Results

### Participant Characteristics and Study Completion

Five participants undergoing elective CABG surgery were enrolled and completed the study procedures. Three participants received Ang-(1–7) (200 μg/kg/day administered subcutaneously) and two received a placebo. Study drug administration began intraoperatively within two hours of chest opening and continued once daily for 21 consecutive days. All participants completed baseline assessments (preoperative screening, 1–15 days prior to CABG) and the Day 21 post-operative evaluation. No participants discontinued study drug prematurely, and there were no study-related withdrawals.

### Participant Demographics

Participant demographics and baseline renal function are summarized in Table 1. Participants were predominantly White and male (80%), with ages ranging from 56 to 75 years.

### Feasibility and Safety

Perioperative administration of Ang-(1–7) was feasible in all treated participants. Study drug infusion was initiated before cardiopulmonary bypass and completed according to protocol in all cases. No infusion interruptions, drug-related adverse events, or hemodynamic instabilities attributable to Ang-(1–7) were observed. Perioperative clinical courses were consistent with expected postoperative recovery following CABG surgery.

No serious adverse events attributable to Ang-(1–7) were observed. Routine perioperative laboratory parameters, including hematologic indices, electrolytes, renal and liver function markers, and coagulation measures, remained clinically stable across participants. No treatment-emergent laboratory abnormalities were identified. Together, these findings support the perioperative safety and feasibility of Ang-(1–7) administration in older adults undergoing CABG surgery.

### Renal Function and Metabolic Safety

Serum creatinine, BUN, and BUN/creatinine ratio remained within clinically acceptable ranges throughout the perioperative period (baseline through day 4 postoperatively) in all participants. Modest perioperative fluctuations were observed, consistent with expected hemodynamic and fluid shifts following CABG surgery. No participant demonstrated a sustained rise in creatinine or laboratory evidence of acute kidney injury. Electrolyte parameters remained stable, and no renal adverse events were attributed to study drug administration.

Renal function was summarized using eGFR (CKD-EPI 2021 race-free equation) derived from serum creatinine. Baseline eGFR ranged from 36 to 102 mL/min/1.73 m², with one participant demonstrating reduced renal function at baseline (eGFR 36) that remained low 4 days post-surgery (lowest observed eGFR 29), while the remaining participants had eGFR values in the mildly reduced to normal range (Table 1).

### Blood Pressure and Hemodynamic Safety

**Figure 1:**
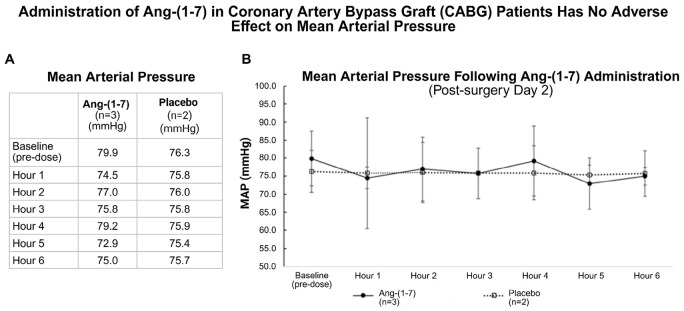
Mean arterial pressure monitoring following perioperative angiotensin-(1–7) administration. **(A)** Mean arterial pressure (MAP) values measured at baseline (pre-dose) and hourly for six hours following study drug administration on postoperative day 2 in coronary artery bypass graft (CABG) patients receiving angiotensin-(1–7) (Ang-(1–7) via subcutaneous injection at a dose of 200 μg/kg; n = 3) or placebo (n = 2). **(B)** Graphical representation of MAP during the same monitoring period. Data are shown as mean ± SEM. MAP remained within clinically acceptable ranges in all participants, with no evidence of treatment-associated hypotension during the observation period.

Post-dosing BP was monitored from baseline through postoperative day 4. To assess potential hemodynamic effects of Ang-(1–7), MAP was measured prior to dosing and hourly for six hours following study drug administration on postoperative day 1. MAP values in Ang-(1–7)–treated participants (n = 3) were comparable to those observed in placebo-treated participants (n = 2), with no evidence of treatment-associated hypotension or hemodynamic instability. These findings indicate that perioperative administration of Ang-(1–7) was well tolerated with respect to cardiovascular safety. Changes in MAP during the monitoring period are shown in Fig. 1.

### Cognitive Outcomes at Day 21 Post-Surgery

Cognitive performance was assessed at baseline during the preoperative screening period (1–15 days prior to CABG) and again at Day 21 following CABG, corresponding to the early post-operative recovery period. Given the small sample size, results are presented descriptively as individual trajectories and group-level trends without formal statistical testing.

### Associative and Recognition Memory

**Figure 2:**
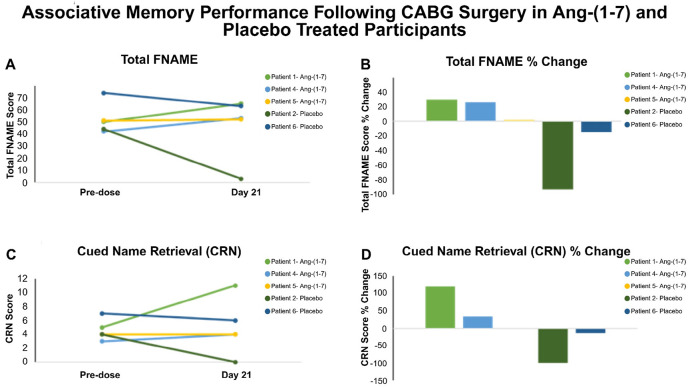
Associative and recognition memory performance following CABG surgery. Cognitive performance was assessed at baseline (pre-dose) and Day 21 after coronary artery bypass graft (CABG) surgery using the Face–Name Associative Memory Examination (FNAME), including Cued Name Retrieval (CRN) performance. Participants received angiotensin-(1–7) (Ang-(1–7)) administered subcutaneously at a dose of 200 μg/kg/day for 21 days (n = 3; Patients 1, 4, and 5) or placebo (n = 2; Patients 2 and 6). The first dose was administered intraoperatively within two hours of chest opening during CABG. Each color represents an individual participant. **(A)** Individual participant trajectories for FNAME total score from baseline to Day 21. **(B)** Percent change from baseline in FNAME total score at Day 21. **(C)** Individual participant trajectories for CRN performance from baseline to Day 21. **(D)** Percent change from baseline in CRN performance at Day 21. Data are presented descriptively without formal statistical testing due to the exploratory nature and small sample size of this case series.

Associative and recognition memory were assessed at baseline and Day 21 using the Face–Name Associative Memory Examination (FNAME), including total FNAME score and Cued Name Recall (CRN) performance. Individual participant trajectories are shown in Fig. 2A and 2C for total FNAME and CRN performance, respectively. Participants receiving Ang-(1–7) generally demonstrated stable or improved performance at Day 21 relative to baseline, whereas placebo-treated participants showed declines on these measures. When expressed as percent change from baseline (Fig. 2B and 2D), Ang-(1–7)–treated participants exhibited positive or modest changes in performance, while placebo-treated participants demonstrated negative changes at the early postoperative time point. These findings suggest relative preservation of associative and recognition memory performance in participants receiving Ang-(1–7) compared with placebo.

Cognitive performance was assessed at baseline (pre-dose) and Day 21 after coronary artery bypass graft (CABG) surgery using the Face–Name Associative Memory Examination (FNAME), including Cued Name Retrieval (CRN) performance. Participants received angiotensin-(1–7) (Ang-(1–7)) administered subcutaneously at a dose of 200 μg/kg/day for 21 days (n = 3; Patients 1, 4, and 5) or placebo (n = 2; Patients 2 and 6). The first dose was administered intraoperatively within two hours of chest opening during CABG. Each color represents an individual participant.. (A) Individual participant trajectories for FNAME total score from baseline to Day 21., (B) Percent change from baseline in FNAME total score at Day 21., (C) Individual participant trajectories for CRN performance from baseline to Day 21., (D) Percent change from baseline in CRN performance at Day 21. Data are presented descriptively without formal statistical testing due to the exploratory nature and small sample size of this case series.

### Other Cognitive Domains

Performance across other assessed domains, including executive function, attention, processing speed, and inhibitory control, did not demonstrate consistent directional trends between groups. Variability across individuals was observed, consistent with the known heterogeneity of perioperative cognitive outcomes following CABG. These measures were therefore interpreted as a supportive context rather than primary indicators of treatment effect.

### Serum Neurofilament Light Chain (NfL)

Serum NfL, a marker of neuroaxonal injury, was measured using a Quanterix Simoa assay. At baseline, CABG patients exhibited higher serum NfL levels compared with age-matched healthy controls, consistent with increased neuroaxonal vulnerability in this population.

**Figure 3:**
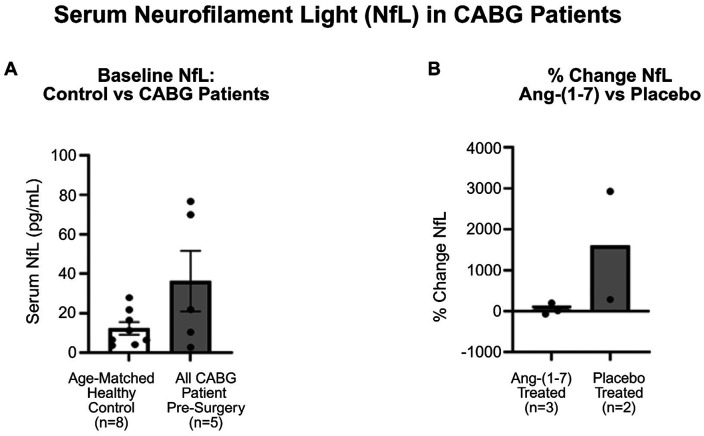
Serum neurofilament light (NfL) levels in CABG patients and controls before and after surgery. **(A)** Baseline serum NfL concentrations measured prior to coronary artery bypass graft (CABG) surgery in age/sex-matched healthy controls (n = 8; white bars) and CABG patients (n = 5; gray bars). **(B)** Individual percent change in serum NfL from baseline to 21 days post-CABG in participants receiving angiotensin-(1-7) (Ang-(1-7); n = 3; white bars) or placebo (n = 2; gray bar). Serum NfL concentrations were quantified using an ultrasensitive single-molecule array (Simoa) assay. Data are shown as individual values with group means ± SEM. Given the exploratory nature of this study and small sample size, results are presented descriptively without formal statistical testing.

As illustrated in Fig. 3A, at Day 21 post-surgery, placebo-treated participants demonstrated an increase in serum NfL relative to baseline. In contrast, participants treated with Ang-(1–7) showed attenuation of the post-operative increase in serum NfL, with levels remaining closer to baseline values (Fig. 3B). These findings suggest a potential protective effect of Ang-(1–7) on perioperative neuroaxonal injury during the early recovery period.

## Discussion and Conclusions

Postoperative cognitive impairment remains a common and clinically significant complication following CABG surgery, with prior studies demonstrating acute and persistent deficits in attention, executive function, and memory following cardiopulmonary bypass (2), (20). Despite decades of investigation, no pharmacologic therapy has been approved to prevent or mitigate this vulnerability. In this exploratory perioperative case series, administration of Ang-(1–7) initiated intraoperatively and continued through early postoperative recovery was feasible, well tolerated, and associated with descriptive signals across cognitive, biomarker, and metabolic domains.

Perioperative Ang-(1–7) administration appeared to be safe in this small sample and was not associated with clinically significant hypotension, renal dysfunction, laboratory abnormalities, or treatment-emergent adverse events. Mean arterial pressure and perioperative renal indices were stable across participants. These findings are consistent with prior clinical studies demonstrating the tolerability of Ang-(1–7) in non-surgical populations (21), (22) and support its feasibility for perioperative investigation in cardiac surgery patients.

Cognitive outcomes in this study were most notable within the domain of associative memory. Participants receiving Ang-(1–7) demonstrated relative preservation of FNAME associative memory performance at 21 days post-CABG, whereas placebo-treated participants exhibited a decline. Associative memory tasks are particularly sensitive to hippocampal–cortical network integrity and have been shown to be vulnerable to vascular, inflammatory, and metabolic insults common in the perioperative period (23). Importantly, no consistent group-level differences were observed across other cognitive domains, underscoring the heterogeneity of postoperative cognitive outcomes and supporting a cautious, domain-specific interpretation rather than a generalized cognitive effect.

These cognitive findings are complemented by observations in serum NfL, a validated blood-based marker of neuroaxonal injury. In this cohort, baseline NfL concentrations were higher in CABG participants than in age-matched controls, consistent with preoperative neuronal vulnerability in patients with advanced cardiovascular disease. At 21 days postoperatively, placebo-treated participants demonstrated increases in NfL, whereas Ang-(1–7)–treated participants exhibited smaller or absent increases. This pattern aligns with recent studies showing that NfL rises following cardiac surgery and is associated with postoperative delirium and early cognitive vulnerability (12), (24). Because circulating NfL levels can be influenced by renal clearance, perioperative kidney function was carefully considered; as renal indices remained stable, the observed differences in NfL are unlikely to be the result of reduced clearance (24). Although exploratory, the directionality of these findings is consistent with broader literature linking perioperative neuroaxonal stress to early postoperative cognitive outcomes (11).

Mechanistically, Ang-(1–7) represents the endogenous counter-regulatory arm of the renin–angiotensin system, acting via the MasR to oppose angiotensin II–mediated oxidative stress, inflammation, and endothelial dysfunction (25), (26). MasRs are expressed on multiple components of the (NVU), including endothelial cells, neurons, and microglia, positioning Ang-(1-7) as a candidate intervention capable of modulating convergent vascular and neural contributors to postoperative cognitive vulnerability (14), (27). Further supporting the biological coherence of the present observations, preclinical studies in models of vascular dementia demonstrate Ang-(1–7)–mediated enhancement of hippocampal synaptic plasticity and protection against ischemia-induced neuronal injury (28), (29), (30), (31).

Several limitations warrant consideration. The small sample size, early termination of enrollment, and descriptive analytic approach preclude definitive conclusions regarding efficacy or generalizability. Cognitive outcomes were assessed at a single early postoperative time point, and longer-term trajectories remain unknown. The study was not designed to disentangle contributions from embolic injury, hypoperfusion, or delirium. Accordingly, these findings should be interpreted strictly as hypothesis-generating.

In conclusion, this exploratory perioperative case series suggests that Ang-(1–7) administration initiated intraoperatively and continued through early recovery is feasible, well-tolerated, and associated with convergent signals of preserved associative memory performance, attenuated postoperative neuroaxonal injury, and metabolic stability following CABG surgery. While these findings do not establish efficacy, they support further investigation of Ang-(1–7) as a mechanism-informed perioperative strategy targeting neurovascular vulnerability in cardiac surgery populations.

## Figures and Tables

**Table 1.** Participant demographics and baseline clinical characteristics. Values are presented as mean (range) for continuous variables and number (percentage) for categorical variables. Baseline renal function was categorized according to estimated glomerular filtration rate (eGFR; CKD-EPI 2021 race-free equation) levels (mL/min/1.73 m^2^).

Participant Demographics Characteristic	Overall (N = 5)
Age, years, mean (range)	64.6 (56–75)
Sex, n (%)	
Male	4 (80%)
Female	1 (20%)
Education, years, mean (range)	14.0 (12–18)
Race, n (%)	
White	5 (100%)
Ethnicity, n (%)	Male
Not Hispanic or Latino	5 (100%)
Baseline renal function (eGFR category*)	Male
Normal or mildly reduced (≥ 60 mL/min/1.73 m^2^)	3 (60%)
Mild–moderate reduction (45–59 mL/min/1.73 m^2^)	1 (20%)
Moderate reduction (30–44 mL/min/1.73 m^2^)	1 (20%)

## Data Availability

The datasets used and/or analyzed during the current study are available from the corresponding author on reasonable request.

## Abbreviations

AEs: Adverse events
Ang-(1–7): Angiotensin-(1–7
BP: Blood pressure
BUN: Blood urea nitrogen
BBB: Blood–brain barrier
CAM: Confusion Assessment Method
CERAD: Consortium to Establish a Registry for Alzheimer’s Disease
CABG: Coronary artery bypass grafting
CRN: Cued Name Recal
eGFR: Estimated glomerular filtration rate
FNAME: Face–Name Associative Memory Examination
MasR: Mas receptor
MAP: Mean arterial pressure
MMSE: Mini-Mental State Examination
NfL: Neurofilament ligh
NVU: Neurovascular unit
RAVLT: Rey Auditory Verbal Learning Test
SAEs: Serious adverse events
TMT: Trail Making Test
